# Yeast Beta-Glucan Supplementation Downregulates Markers of Systemic Inflammation after Heated Treadmill Exercise

**DOI:** 10.3390/nu12041144

**Published:** 2020-04-19

**Authors:** Hannah A. Zabriskie, Julia C. Blumkaitis, Jessica M. Moon, Brad S. Currier, Riley Stefan, Kayla Ratliff, Patrick S. Harty, Richard A. Stecker, Karolina Rudnicka, Ralf Jäger, Michael D. Roberts, Kaelin Young, Andrew R. Jagim, Chad M. Kerksick

**Affiliations:** 1 Department of Kinesiology, Towson University, Towson, MD 21252, USA; hzabriskie@towson.edu; 2 Exercise and Performance Nutrition Laboratory, School of Health Sciences, Lindenwood University, 209 S. Kingshighway, St. Charles, MO 63301, USA; jcb178@lindenwood.edu (J.C.B.); jmoon@lindenwood.edu (J.M.M.); currierB@mcmaster.ca (B.S.C.); rstefan1@niu.edu (R.S.); kr561@lindenwood.edu (K.R.); rstecker@lindenwood.edu (R.A.S.); 3 Energy Balance and Body Composition Laboratory, Department of Kinesiology & Sport Management, Texas Tech University, Lubbock, TX 79409, USA; Patrick.Harty@ttu.edu; 4 Department of Immunology and Infectious Biology, Faculty of Biology and Environmental Protection, University of Lodz, 90-136 Lodz, Poland; karolina.rudnicka@biol.uni.lodz.pl; 5 Increnovo LLC, Milwaukee, WI 53202, USA; ralf.jaeger@increnovo.com; 6 School of Kinesiology, Auburn University, Auburn, AL 36849, USA; mdr0024@auburn.edu; 7 Department of Cell Biology and Physiology, Edward Via College of Osteopathic Medicine, Auburn University, Auburn, AL 36849, USA; kyoung@auburn.vcom.edu; 8 Sports Medicine, Mayo Clinic Health System, Onalaska, WI 54650, USA; jagim.andrew@mayo.edu

**Keywords:** immunity, inflammation, supplementation, immune, yeast, carbohydrate, prebiotic, fiber, endurance, hot, humid, supplementation, muscle damage, recovery, yeast beta-glucan

## Abstract

Aerobic exercise and thermal stress instigate robust challenges to the immune system. Various attempts to modify or supplement the diet have been proposed to bolster the immune system responses. The purpose of this study was to identify the impact of yeast beta-glucan (*Saccharomyces cerevisiae*) supplementation on exercise-induced muscle damage and inflammation. Healthy, active men (29.6 ± 6.7 years, 178.1 ± 7.2 cm, 83.2 ± 11.2 kg, 49.6 ± 5.1 mL/kg/min, *n* = 16) and women (30.1 ± 8.9 years, 165.6 ± 4.1 cm, 66.7 ± 10.0 kg, 38.7 ± 5.8 mL/kg/min, *n* = 15) were randomly assigned in a double-blind and cross-over fashion to supplement for 13 days with either 250 mg/day of yeast beta-glucan (YBG) or a maltodextrin placebo (PLA). Participants arrived fasted and completed a bout of treadmill exercise at 55% peak aerobic capacity (VO_2Peak_) in a hot (37.2 ± 1.8 °C) and humid (45.2 ± 8.8%) environment. Prior to and 0, 2, and 72 h after completing exercise, changes in white blood cell counts, pro- and anti-inflammatory cytokines, markers of muscle damage, markers of muscle function, soreness, and profile of mood states (POMS) were assessed. In response to exercise and heat, both groups experienced significant increases in white blood cell counts, plasma creatine kinase and myoglobin, and soreness along with reductions in peak torque and total work with no between-group differences. Concentrations of serum pro-inflammatory cytokines in YBG were lower than PLA for macrophage inflammatory protein 1β (MIP-1β) (*p* = 0.044) and tended to be lower for interleukin 8 (IL-8) (*p* = 0.079), monocyte chemoattractment protein 1 (MCP-1) (*p* = 0.095), and tumor necrosis factor α (TNF-α) (*p* = 0.085). Paired samples *t*-tests using delta values between baseline and 72 h post-exercise revealed significant differences between groups for IL-8 (*p* = 0.044, 95% Confidence Interval (CI): (0.013, 0.938, *d* = −0.34), MCP-1 (*p* = 0.038, 95% CI: 0.087, 2.942, *d* = −0.33), and MIP-1β (*p* = 0.010, 95% CI: 0.13, 0.85, *d* = −0.33). POMS outcomes changed across time with anger scores in PLA exhibiting a sharper decline than YBG (*p* = 0.04). Vigor scores (*p* = 0.04) in YBG remained stable while scores in PLA were significantly reduced 72 h after exercise. In conclusion, a 13-day prophylactic period of supplementation with 250 mg of yeast-derived beta-glucans invoked favorable changes in cytokine markers of inflammation after completing a prolonged bout of heated treadmill exercise.

## 1. Introduction

Strenuous exercise elicits a cascade of acute physiological responses that can temporarily suppress innate immune system activity [1,2,3]. These acute changes include reduction in blood cell count and decreases in natural killer cells, monocyte, and T-cell activity as well as increases in several pro-inflammatory cytokines and chemokines, soreness, and expressions of muscle damage mediators [4,5,6]. Exercising in the heat appears to exacerbate these physiological responses, especially gastrointestinal distress, which often accompanies acute-phase immune cell activity [7]. Thus, individuals engaging in regular strenuous exercise are often at higher risk to develop upper respiratory infections and other infections that may require a cessation of exercise or lead to reductions in training quality, consistency, and overall performance. In addition, intense exercise can cause exercise-induced muscle damage (EIMD), which is characterized by intramuscular inflammation, soreness, and acute reductions in muscle function [8]. The intramuscular inflammation following EIMD is a tightly coordinated and dynamic process that eventually leads to adaptive remodeling and return to homeostasis [9]. Various cell types, including neutrophils, macrophages, mast cells, eosinophils, cluster of differentiation 8 (CD8) glycoproteins and T-regulatory lymphocytes, fibro-adipogenic progenitors, and pericytes, help to facilitate muscle tissue repair [9]. In general, the adaptive process following EIMD is made up of two stages: An acute phase characterized by increased neutrophil and pro-inflammatory macrophage activity, and a second stage during which regulatory macrophages as well as lymphocytes are involved. Of these cells, macrophages that infiltrate the site of muscle inflammation seem to be the most potent regulators of both stages of the muscle repair process [10].

To date, a variety of nutritional interventions and dietary supplements have been suggested to attenuate the detrimental effects of strenuous exercise on immune function [11,12]. For example, it appears that carbohydrate supplementation may decrease exercise-induced immunosuppression by reducing the observed disturbances in white blood cell counts as well as the expression of certain pro-inflammatory cytokines [5,13]. Similarly, regular supplementation of nondigestible carbohydrates, such as beta-glucans found within the cellular membranes of yeast, fungi, and oats, can play a vital role in supporting innate immune system function. The mechanism of immunoregulation mediated by beta-glucans depends on their interaction with immune cells localized in the intestines, which recognize these oligosaccharides and induce local and systemic regulatory responses. Beyond obvious structural differences, beta-glucans, unlike carbohydrates, are not absorbed in the small intestine and pass into the large intestine. In vitro as well as in vivo evidence has shown that macrophages regularly exposed to yeast beta-glucans have higher phagocytic as well as cytokine activity and that their responses are characterized by anti-inflammatory patterns involved in the regeneration process [14,15]. In addition, it appears that yeast beta-glucan can improve immune defenses to prevent or shorten the duration of common colds [16,17] as well as enhance the immune system activity following exercise [17]. 

The molecular structure of beta-glucans appears to be a determining factor in their efficacy. For example, the beta-glucans contained within oats and barley have a linear structure, unlike those found in mushrooms and yeast, which have a more branched-like structure that elicits greater increases in biological activity [4]. Prior studies have indicated that oat beta-glucans may not impact immune changes or the onset of upper respiratory infections [18], and that unlike yeast beta-glucans, fungal and plant-derived beta-glucans do not induce trained immunity in macrophages [19]. In this respect, supplementation with yeast beta-glucans may afford an improved level of protection from infection during periods of rigorous physical activity or exercise training. Indeed, previous research has documented the ability of yeast-derived beta-glucans to impact immunity and prevent the onset of colds and other acute infections as well as improve the overall management of such illnesses [20,21,22]. In addition, baker’s yeast beta-glucan supplementation has been shown to reduce the onset of upper respiratory tract infections relative to placebo over a four-week period after running a marathon [23]. Likewise, McFarlin et al. [16] reported that consumption of a yeast-based beta-glucan was associated with a 37% reduction in cold and flu symptoms after a marathon. The authors also reported that salivary expression of immunoglobulin A was increased by approximately 32% relative to placebo in subjects who consumed yeast beta glucan prior to cycling exercise in a hot and humid environment. Similarly, Carpenter and colleagues [4] recruited 60 recreationally active men and women to complete a 10-day prophylactic supplementation period with either yeast-derived beta-glucans or placebo prior to a bout of cycling exercise in a hot and humid environment, and reported that yeast-derived beta-glucans may alter the production of selected cytokines in response to heat and exercise. Finally, McFarlin and investigators [24] recruited healthy, recreationally active volunteers to complete a 10-day supplementation period with 250 mg of a yeast beta-glucan or a placebo before exercising for 90 min in a hot and humid environment. Blood samples were collected before and after the exercise sessions and analyzed for changes in cytokines and immune cell antigens and markers. The authors concluded that yeast beta-glucan supplementation may decrease the susceptibility of opportunistic infections after strenuous exercise.

The ability to perform, tolerate, and recover from intense physical exertion is crucial for exercising individuals. Though a variety of nutritional and supplementation interventions have been suggested to improve these parameters, further information is required in many areas. In this respect, no previous research has examined the impact of baker’s yeast beta-glucan on muscle function, mood, affect, and various blood-based markers of muscle damage following intense exercise. Therefore, the purpose of this study was to examine the efficacy of yeast beta-glucan supplementation to regulate exercise-induced immunosuppression, muscle damage, muscle function, and mood state after an extended bout of treadmill exercise in a hot and humid environment.

## 2. Materials and Methods 

### 2.1. Experimental Design

**Figure 1:**
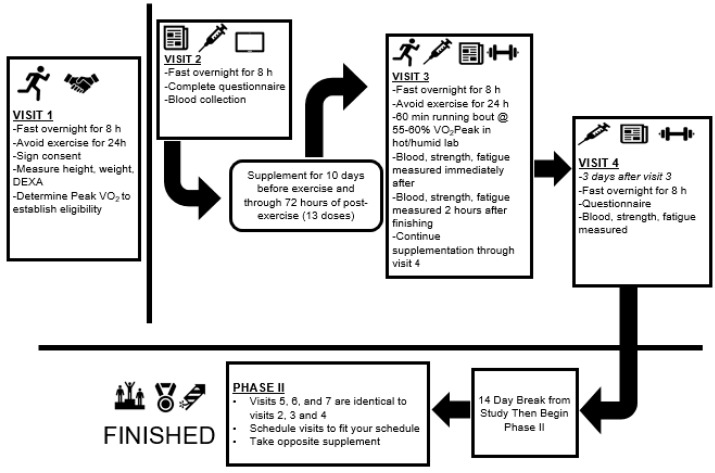
Research design overview. DEXA: dual energy x-ray absorptiometry; VO_2Peak_: peak aerobic capacity

Recreationally active males and females participated in this randomized, double-blind, placebo-controlled, cross-over study design. Participants were told to report to the laboratory in the morning after observing an 8–10-hour fast while maintaining adequate hydration. Additionally, participants were asked to abstain from exercise and avoid alcohol and caffeine consumption for 24 h prior to every study visit. All participants first completed an initial eligibility visit (Visit 1), during which a comprehensive medical history, training history, body composition assessment, and peak aerobic capacity test were performed. Peak aerobic capacity was assessed as the highest value of oxygen consumption (VO_2Peak_) measured during this test and was used to finalize study eligibility. The remainder of the study consisted of two identical patterns of testing (Visits 2–4 were identical to Visits 5–7) with the exception of what supplementation regimen was followed (See Figure 1). During visit 2, each participant reported to the laboratory for a baseline assessment during which questionnaires regarding mood state and soreness were completed and a blood sample was obtained. Participants were fitted and familiarized to the use of the isokinetic dynamometer (Biodex System 3, Biodex Medical Systems, Inc., Shirley, New York, USA) to ensure all subjects were competent in the assessments that would be used later in the study. An isokinetic dynamometer was used to complete assessments of maximal isometric force production, dynamic force and torque production, and muscular fatigue rates of the dominant quadriceps. At the completion of Visit 2, study participants were provided with their supplement and instructed to begin their supplementation so they would consume their 11th dose on the same morning as Visit 3, when the exercise trial was complete. Participants continued to supplement daily through their 72-hour post-exercise testing session (Visit 4) for a total of 13 days of supplementation. On the morning of Visit 3, participants reported to the laboratory after following identical pre-study instructions as previous visits (fasting, abstention from caffeine and exercise). Study participants again completed questionnaires regarding soreness and the Profile of Mood State (POMS), provided a blood sample, and performed muscular force and fatigue assessments using an isokinetic dynamometer immediately before, immediately after exercise, and two hours after termination of the exercise bout. In the 72 h following the exercise visit (i.e., days 11–13 of supplementation), participants were instructed to continue supplementing but to avoid all exercise. The participants returned to the laboratory for Visit 4, which consisted of soreness and POMS questionnaires, venous blood collection, and muscular force and fatigue testing. A washout period lasting a minimum of 14 days and a maximum of 21 days separated each phase of the crossover. After the washout phase, participants completed study Visits 5–7 which were identical in sequence and composition to Visits 2–4 while consuming the alternative supplement. All research procedures were approved by the Institutional Review Board (IRB) prior to the enrollment of participants. This study was retrospectively registered with a International Standard Randomized Control Trials Number (ISRCTN, www.isrctn.com) on 2 April 2020 (ISRCTN 16680414). 

### 2.2. Participants

**Figure 2:**
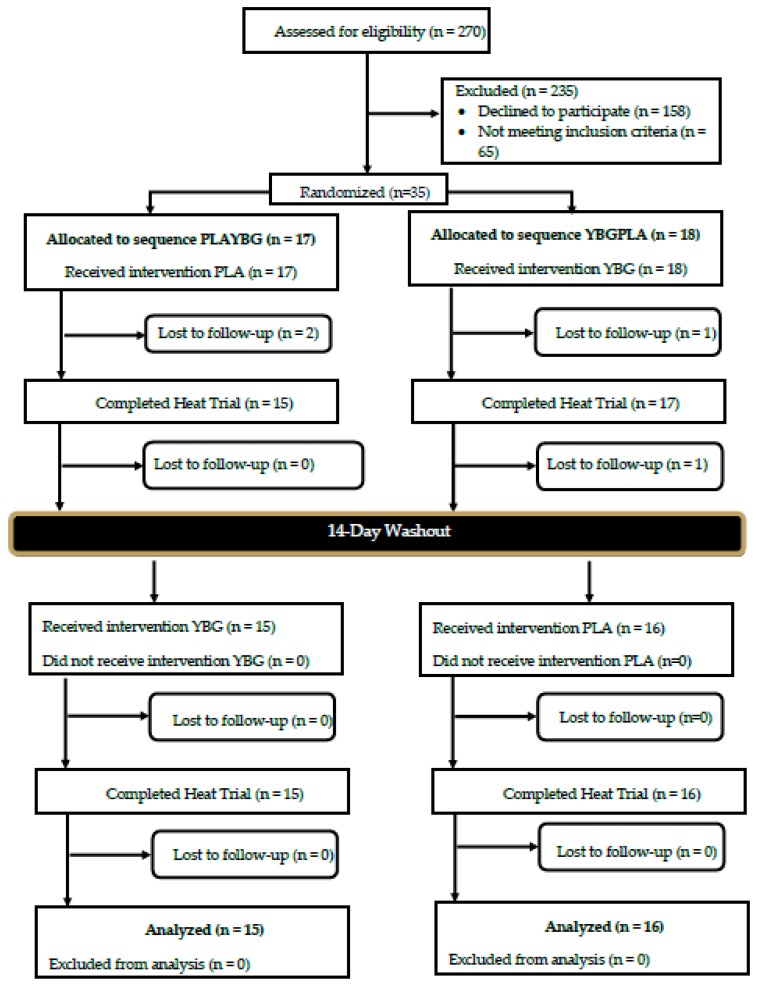
Consolidation Standards of Reporting Trials (CONSORT) Diagram. PLA: Placebo; YBG: Yeast Beta-Glucan; PLAYBG: participant first received PLA followed by YBG; YBGPLA: participant first received PLA followed by YBG.

A Consolidation Standards of Reporting Trials (CONSORT) diagram for all study recruitment, randomization, and project completion is provided in Figure 2. Data collection commenced in July 2018 and was completed in June 2019. A total of 16 male (29.6 ± 6.7 years, 178.1 ± 7.2 cm, 83.2 ± 11.2 kg, 21.2 ± 4.4% fat, 49.6 ± 5.1 mL/kg/min) and 15 female (30.1 ± 8.9 years, 165.6 ± 4.1 cm, 66.7 ± 10.0 kg, 29.6 ± 5.3% fat, 38.7 ± 5.8 mL/kg/min) participants successfully completed the study protocol (see Table 1 for participant characteristics). Prior to participation, participants provided their signed informed consent using an IRB-approved consent document (Protocol # IRB-19-L0035, approval date: 18 December 2018). Using data outlined from previous investigations [25,26], a power analysis indicated that, if moderate effects (*d* = 0.50) of changes in cytokine and muscle function were found at a power of 0.80, a sample size of approximately 30 participants would be needed.

Inclusion criteria included age (18–50 years) and routine physical activity (reported performing some form of aerobic exercise at least twice per week for the last 12 months). Individuals who were currently being treated for or were diagnosed with a cardiac, respiratory, circulatory, musculoskeletal, metabolic, immune, autoimmune, psychiatric, hematological, neurological, or endocrinological disorder or disease were excluded from the study. Finally, any females who were pregnant or breast-feeding were also excluded. Participants who did not meet any exclusion criteria were scheduled to complete the VO_2Peak_ procedure to finalize their eligibility. Final eligibility was determined via the VO_2Peak_ assessment, with 28 of 31 participants having a VO_2Peak_ between the 50th and 80th percentile for their age and gender [27]. The remaining three participants had VO_2Peak_ capacities within 1.5 mL/kg/min of the required cut-off values.

### 2.3. Procedures

#### 2.3.1. Body Composition

During Visit 1, body composition was quantified using a dual-energy X-ray absorptiometry (DEXA) scan (Hologic QDR Discovery A, Hologic, Inc., Bedford, MA, USA) immediately prior to the VO_2Peak_ assessment. Before the scan, height and weight were measured for every participant without shoes and in minimal clothing using a self-calibrating digital scale (Model # BWB-627A Class III, Tanita, Tokyo, Japan) and a standardized wall-mounted stadiometer (Model # HR-200, Tanita, Tokyo, Japan). Positioning of participants and manual analysis of the DEXA scans were completed by a trained research assistant. The DEXA was calibrated daily according to manufacturer recommendations and each scan was analyzed using the provided software (Hologic APEX Software, Version 4.5.3, Hologic Inc., Bedford, MA, USA) with the National Health and Nutrition Examination Survey (NHANES) analysis approach employed. Body composition metrics were recorded for descriptive purposes only.

#### 2.3.2. VO_2Peak_ Assessment

To determine eligibility, participants completed a VO_2Peak_ assessment using a metabolic cart (True Max 2400 Metabolic Measurement System, ParvoMedics, Sandy, UT, USA), which was calibrated within 2% of the previous calibration value each day. A graded, staged treadmill protocol, adapted from the validated protocol published by Camic et al. [28] was used. The test began with a one-minute warm-up at 3 mph and 1% grade. After completion of the warm-up, the protocol utilized two-minute stages starting at 5 mph and 1% grade, and increasing the speed by 1 mph at each stage change up to 9 mph. After completion of the 9-mph stage, the grade increased to 2.5% and increased by an additional 2.5% every two minutes. Participants were instructed to run to exhaustion. Heart rate was monitored throughout the test (Polar FT1, Polar, Kempele, Finland) and was recorded, along with rating of perceived exertion (RPE), during the last 30 seconds of each stage. Once VO_2Peak_ was achieved at the point of volitional fatigue, participants were instructed to walk at 1.5 mph until they felt they had recovered sufficiently. Every eligible participant demonstrated a peak respiratory exchange ratio (RER) of at least 1.00 during the protocol.

#### 2.3.3. Blood Collection and Analysis

Participants provided a venous blood samples (~20 mL) before supplementation, pre-exercise, immediately post-exercise, two hours post-exercise, and 72 h post-exercise. For each collection, two Vacutainer tubes containing dipotassium (K2) and ethylenediaminetetraacetic acid (EDTA) and one serum-separation tube were used. Upon collection, all blood samples were gently inverted 10 times and allowed to set at room temperature. One of the K2-EDTA-containing tubes and the serum separation tube were centrifuged at 20 °C for 15 min at 2060 g (MegaFuge XFR, Thermo Fisher Scientific, Waltham, MA, USA) for the preparation of plasma and serum, respectively. After centrifugation, serum was extracted and transferred into a sterile transfer container while plasma was extracted from the previously centrifuged K2-EDTA tube and aliquoted (800 µL) into separate microcentrifuge tubes. The aliquoted plasma samples were subsequently stored at –80 °C until later cytokine, creatine kinase, and myoglobin analysis. The processed serum and remaining whole-blood EDTA sample were stored in a chilled compartment and shipped to a commercial diagnostic laboratory (Quest Diagnostics, St. Louis, MO, USA) the same day for analysis of a comprehensive metabolic panel and complete blood count with platelet differentials.

#### 2.3.4. Biochemical Analysis

Plasma creatine kinase was analyzed in triplicate using a standard absorbance-based, enzymatic muscle creatine kinase assay (PointeScientifc, Canton, MI, USA) using an automated chemistry analyzer (Chemwell 2910, Awareness Technologies, Palm City, FL, USA) according to manufacturer recommendations. Any samples flagged as abnormally low or high were reanalyzed to confirm the result. Plasma myoglobin concentration was determined in duplicate using a commercially available ELISA assay kit (Immunology Consultants Laboratory, Inc., Portland, OR, USA). Absorbance values were determined using an automated microplate reader (BioTek EPOCH, Winooski, VT, USA). Sample values were determined using a standard curve on each ELISA plate. Plasma concentrations of interleukin-1β (IL-1β), interleukin-2 (IL-2), interleukin-4 (IL-4), interleukin-5 (IL-5), interleukin-6 (IL-6), interleukin-7 (IL-7), interleukin-8 (IL-8), interleukin-10(IL-10), interleukin-12 (IL-12), interleukin-13 (IL-13), interleukin-17 (IL-17,) granulocyte colony-stimulating factor(G-CSF), tumor necrosis factor α (TNFα), granulocyte-macrophage colony-stimulating factor (GM-CSF), interferon-γ (IFNγ), monocyte chemoattractant protein-1 (MCP-1), and macrophage inflammatory protein-1β (MIP-1β) cytokines were determined using a commercially available 96-well multiplex assay (Bio-Plex Pro Human Cytokine 17-plex, Bio-Rad, Hercules, CA, USA) following the manufacturer’s procedures and read using a Luminex FLEXMAP 3D system (Luminex Corp., Austin, TX, USA). Notably, only IL-6, IL-8, IFNγ, MCP-1, MIP-1β, and TNF-α concentrations are reported herein as other target levels resulted in concentrations below the sensitivity of the assay. All samples were analyzed in duplicate and resulted in an average intra-assay percent of coefficient of variability (%CV) of 5.3% (range of 4.51–6.28%) for the aforementioned targets. All results are expressed as picograms (pg)/mL based on standard curves.

#### 2.3.5. Muscular Force and Fatigue Assessments

After obtaining a blood sample, participants were instructed to perform a standard dynamic warm-up consisting of simple body weight exercises that took approximately five minutes to complete. Participants then performed two musculoskeletal assessments on a Biodex System 3 Isokinetic Dynamometer (Biodex Medical Systems, Inc., Shirley, NY, USA). The first test was a maximal voluntary isometric contraction of the knee extensors performed at 60° of knee flexion. Three separate five-second maximal contractions were completed with approximately 30 seconds between repetitions. The peak force generated from all three repetitions was used for data analysis. Five minutes of rest was given between each test. The next test was completed according to the procedures of Thorstensson [29] and required participants to complete 50 concentric-only repetitions of the knee extensors at 180 degrees per second. Isokinetic peak torque and fatigue index were recorded and analyzed. These assessments were performed at four time points: Before exercise, immediately post-exercise, two hours post-exercise, and 72 h post-exercise.

#### 2.3.6. Assessment of Mood State and Soreness

At five time points during each phase of the study, participants completed electronic versions of the POMS questionnaire and a 100-mm visual analog scale to quantify muscle soreness anchored with 0 is ‘No Soreness At All’ and 100 is ‘Extreme, Debilitating Soreness.’ The five time points were as follows: Baseline, before exercise, immediately post-exercise, two hours post-exercise, and 72 h post-exercise. All 65 items on the POMS were scored and summed into profiles for tension, anger, vigor, fatigue, depression, and confusion. In addition, a total mood disturbance score was calculated by summing all categories and subtracting the vigor score.

#### 2.3.7. Supplementation

In a double-blind manner, a beta-glucan supplement (Yestimun®) derived from *Saccharomyces cerevisiae* or a maltodextrin placebo was distributed in identical opaque capsules to all participants. Because of the cross-over design utilized in this study, all participants ingested both beta-glucan and placebo in different phases of the study. The order of administration was randomized separately for each gender using the flip of a coin. Participants were assigned to either placebo followed by beta-glucan (PLA-YBG sequence) or beta-glucan followed by placebo (YBG-PLA sequence).For every other male or female that joined the study, a coin was flipped to determine their supplement order, and the next person of the same gender to join the study received the opposite order. After order was determined for two individuals using a single coin flip, a coin was flipped again to determine order for the next two individuals of a given gender. This method allowed for counterbalancing within each gender and resulted in 17 participants assigned to the PLA-YBG sequence while 18 participants were assigned to the YBG-PLA sequence. On a daily basis, participants were instructed to ingest one capsule, containing 250 mg of beta-glucan (Yestimun®, Leiber GmbH, Germany) or a corn maltodextrin (Maltrin M100, Grain Processing Corporation Muscatine, IA, USA) as a placebo. The study materials were prepared by Mango-Humphries Labs, Inc. (Tigard, OR, USA) using hard capsules of vegetable origin (CapsCanada, Tecumseh, ON, Canada, product code KK01-1002). Participants consumed each supplement for a total of 13 days, with baseline visits occurring on or before the first day of supplementation while the bout of heated treadmill exercise was completed on the 11th day of supplementation (Figure 1). Participants were instructed to avoid ingestion of the supplement within two hours of a meal and approximately at the same time each day. In the case of a missed dose, participants were instructed to consume the missed dose as soon as possible. For some, this meant taking two doses at the same time. No participant reported any adverse effects to the supplementation protocol.

#### 2.3.8. Exercise Bouts

Prior to each exercise bout, participants were assessed for hydration by providing a mid-flow urine sample using a handheld urine refractometer. Any participant who had a urine-specific gravity greater than 1.020 was required to hydrate until they could provide a sample demonstrating that they were adequately hydrated. Body weight without shoes was also obtained immediately prior to the exercise bout. Oxygen consumption was intermittently measured before exercise began and every 15 min (for 3 min) during the exercise bout using a metabolic cart. RPE and heart rate were likewise recorded every 10 min during exercise. Participants were allowed to ingest as much water as they desired during Visit 2 but were required to consume the same amount of water for their subsequent exercise bout. Each exercise bout consisted of graded walking at 3.0 mph and the grade predicted to elicit approximately 55% VO_2Peak_ based upon the metabolic equations published by the American College of Sports Medicine (ACSM) [30]. All exercise was performed on a treadmill (Woodway DESMO-EVO, Woodway USA, Waukesha, WI, USA) in a heated and humidified (35–40 °C, 40–45% relative humidity (RH)) chamber (See Table 2). Each bout could last up to 60 min. The exercise was terminated (and duration recorded) if core temperature reached 39.2 °C or significant symptoms (e.g., dizziness or nausea) developed which inhibited the completion of the bout or participant requested to stop. In a manner similar to the approach employed by Carpenter et al. [4], if the bout was terminated early, the second exercise bout was matched in time. Immediately upon completion of the exercise bout, participants were relocated to the normothermic laboratory where they cooled down by walking on a treadmill at 3.0 mph for three minutes. Upon immediate completion of the cool down, participants provided another urine sample and were weighed nude.

#### 2.3.9. Safety Measures: Temperature Monitoring and Rehydration

During each baseline visit (Visits 1 and 4), participants were provided with an ingestible body temperature sensor (CorTemp Ingestible Core Body Temperature Sensor, HQ Inc., Palmetto, FL, USA). Three to four hours prior to each exercise bout, participants ingested the temperature sensor, which would enable researchers to monitor core temperature during exercise without any interruption in completion of the exercise bout. Temperature was recorded at least every 10 min during the exercise bout; however, temperature was taken with increasing frequency if core temperature became greater than 38.5 °C. Participants were rehydrated over the next two hours using 1.5 times the measured body mass loss in kilograms, similar to the methods of Stasiule and colleagues [31]. The rehydration volume was divided into four equal doses given every 30 min. At two hours post-exercise, participants provided a final urine sample to demonstrate that hydration had been restored.

#### 2.3.10. Dietary Control

In the 24 h prior to the first exercise bout (Visit 2), participants were instructed to keep a log of all food and drink using a commercially available food logging application (MyFitnessPal, Under Armour, Baltimore, MD, USA). Participants were instructed to replicate this diet in the 24 h preceding Visit 5 and verbal confirmation was obtained before proceeding to any additional procedures during Visit 5.

### 2.4. Statistical Analysis

All analyses were completed using Microsoft Excel and the Statistical Package for the Social Sciences (v26, SPSS Inc., Chicago, IL, USA). Prior to analysis, data were examined for missing values using Little’s Test for Missing Completely at Random (MCAR). Serum and whole blood clinical markers and soreness values were missing at least one data point (*n* ≤ 10 data points) and were identified as MCAR using this method. Multiple imputation by fully conditional specification was employed to replace missing values for all serum and whole-blood clinical markers as well as for soreness values. The results of 10 imputations were pooled and used for all subsequent analyses. Two data points were determined to be missing from the cytokine data. Both missing data points were from different treatment conditions and at different time points. Consequently, these missing data points were replaced using an intent-to-treat approach with the last observed data point being brought forward. Following the completion of missing data replacement, descriptive statistics were calculated and are presented as mean ± standard deviation. Before any statistical tests were completed, data were analyzed for normality and all non-normal data were transformed prior to analysis. Non-normal data were first transformed using log, then square root, and finally cubic to establish normality. Transformed data were then used for all statistical analysis, but raw data were used for presentation of data in all figures and tables. For all statistical tests, a significance level of α= 0.05 was utilized as the basis for statistical determinations. A statistical tendency or trend was considered if an observed p-value was between 0.051 and 0.100. Mixed (group (2) × time (4)) factorial analysis of variance (ANOVA) with repeated measures on time was used to assess the impact of supplementation on all outcome variables. When significant main effects for time were identified, single-factor repeated measures ANOVAs were performed with time as a within-subjects factor. If the sphericity assumption was violated, either Greenhouse–Geisser or Huynh–Feldt corrections were applied, depending on the estimated epsilon value. Bonferroni post hoc corrections were used to control the familywise error rate for all pairwise comparisons. To further decompose the ANOVA model, change scores from baseline were computed and compared at each time using paired samples *t*-tests at each individual time point. The threshold of significance was set at α = 0.05.

## 3. Results

### 3.1. Physiological Stress

All indicators of physiological stress throughout the exercise bout are provided in Table 2. Paired samples t-test were computed for the data collected at each time point throughout exercise to assess differences between groups for all collected heart rate, rate of perceived exertion (RPE), oxygen consumption (VO_2_), and core temperature values. No statistically significant changes (*p* > 0.05) were observed between groups for any variables (see Table 2).

### 3.2. Cytokine and Chemokine Expression

**Figure 3:**
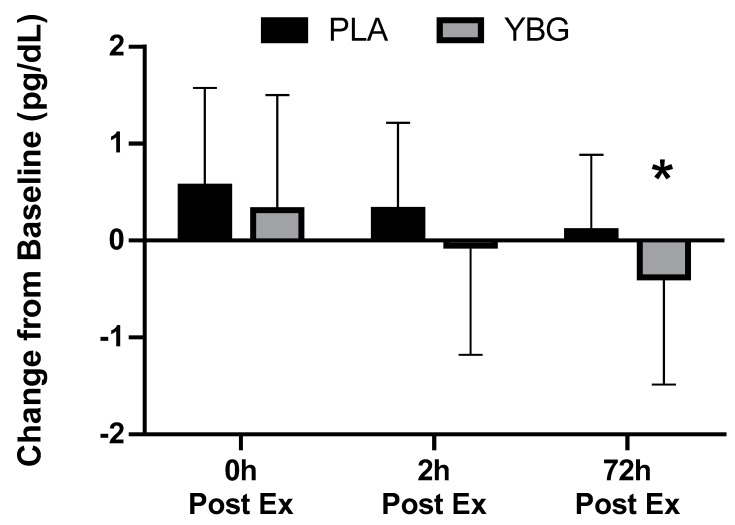
Changes in interleukin-8 (IL-8) expression from baseline for YBG and PLA after 13 days of supplementation and heated treadmill exercise. Data are presented as means ± SD. The 4 × 2 mixed factorial ANOVA revealed a group × time interaction effect of *p* = 0.079 along with a significant main effect across time (*p* < 0.001). Changes from baseline were calculated and compared using paired samples t-test. At the 72-hour time point, the changes observed in YBG were significantly different (mean difference ± SD: 0.48 ± 1.26, 95% confidence interval (CI) on difference: (0.013, 0.938), *p* = 0.044) than the changes observed in PLA. Percent changes from baseline for PLA were +32.2%, +18.4%, and +5.8% while % changes from baseline for YBG were +15.0%, –2.6%, and –18.4% after 0, 2, and 72 h post-exercise, respectively. * indicates statistically significant difference in paired samples t-test at 72 h timepoint.

**Figure 4:**
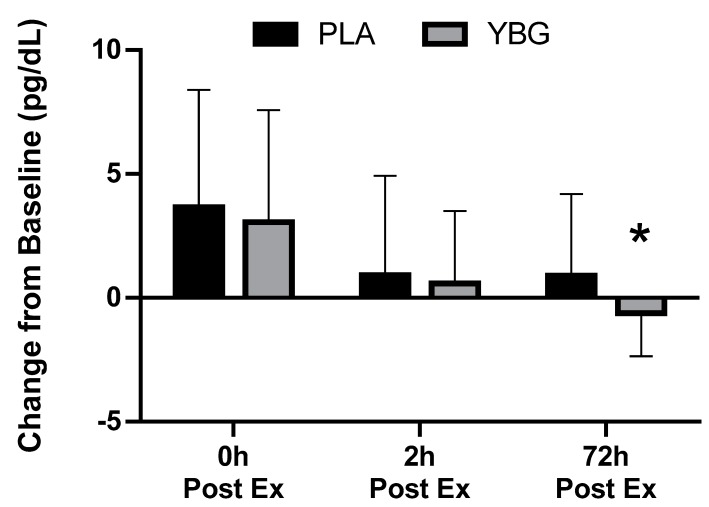
Changes in monocyte chemoattractant protein-1 (MCP-1) expression from baseline for YBG and PLA after 13 days of supplementation and heated treadmill exercise. Data are presented as means ± SD. The 4 × 2 mixed factorial ANOVA revealed a group × time interaction effect of p = 0.095 along with a significant main effect across time (*p* < 0.001). Changes from baseline were calculated and compared using paired samples *t*-test. At the 72-hour time point, the changes observed in YBG were significantly different (mean difference ± SD: 1.51 ± 3.89, 95% CI on difference: (0.087, 2.942), *p* = 0.038) than the changes observed in PLA. Percent changes from baseline for PLA were +43.0%, +10.7%, and +10.5% while % changes from baseline for YBG were +33.0%, +6.3%, and –6.8% after 0, 2, and 72 h post-exercise, respectively. * indicates statistically significant difference in paired samples t-test at 72 h timepoint.

**Figure 5:**
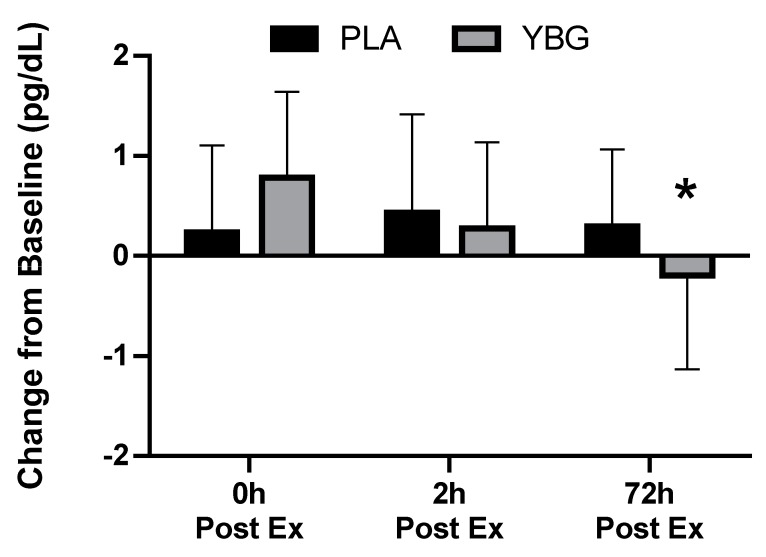
Changes in macrophage inflammatory protein-1β (MIP-1β) expression from baseline for YBG and PLA after 13 days of supplementation and heated treadmill exercise. Data are presented as means ± SD. The 4 × 2 mixed factorial ANOVA revealed a group × time interaction effect of *p* = 0.044 along with a significant main effect across time (*p* < 0.001). Changes from baseline were calculated and compared using paired samples t-test. At the 72-hour time point, the changes observed in YBG were significantly different (mean difference ± SD: 0.49 ± 0.99, 95% CI on difference: (0.13, 0.85), *p* = 0.010) than the changes observed in PLA. Percent changes from baseline for PLA were +14.1%, +25.7%, and +17.6% while % changes from baseline for YBG were +3.7%, +12.9%, and –9.1% after 0, 2, and 72 h post-exercise, respectively. * indicates statistically significant difference in paired samples t-test at 72 h timepoint.

**Figure 6:**
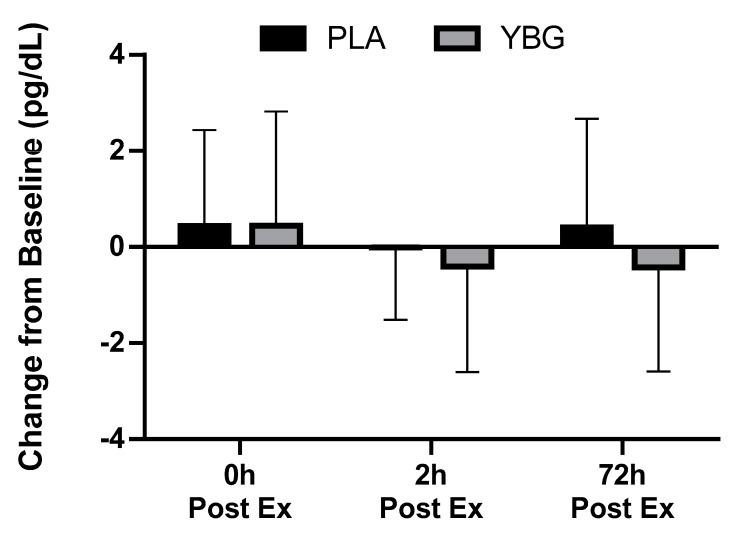
Changes in tumor necrosis factor α (TNF-α) expression from baseline for YBG and PLA after 13 days of supplementation and heated treadmill exercise. Data are presented as means ± SD. The 4 × 2 mixed factorial ANOVA revealed a group × time interaction effect of *p* = 0.085. Changes from baseline were calculated and compared using paired samples t-test. At the 72-hour time point, the changes observed in YBG tended to be different (mean difference ± SD: 0.83 ± 2.71, 95% CI on difference: (−0.17, 1.83), *p* = 0.10) than the changes observed in PLA. Percent changes from baseline for PLA were +7.4%, –0.2%, and +6.9% while % changes from baseline for YBG were +6.8%, –6.1%, and –6.5% after 0, 2, and 72 h post-exercise, respectively.

Prior to and in response to the heated exercise, plasma concentrations of IL-1β, IL-6, IL-12, GM-CSF, TNF-α, IL-2, IL-7, IL-13, IFNγ, IL-4, IL-8, IL-17, MCP-1, IL-6, IL-10, G-CSF, and MIP-1β were determined. Of the 17 cytokines analyzed, six were consistently detected in the blood at concentrations that allowed for statistical analysis. A significant group x time (GxT) interaction effect was observed (*p* = 0.044) for MIP-1β. PLA was increased by 17.9% while YBG was decreased by 8.9% when assessed 72 h after the exercise bout. Changes in IL-8 (GxT, *p* = 0.08), MCP-1 (GxT, *p* = 0.10), and TNF-α (GxT, *p* = 0.09) all exhibited a statistical tendency (defined as *p* = 0.051–0.10) to be different between groups. When each group’s effects were examined individually in comparison to their respective baseline levels, IL-8 and MCP-1 in the YBG group were significantly reduced from baseline after 72 h while changes in PLA demonstrated increases from baseline. Similarly, TNF-α levels portrayed non-significant increases in the PLA group while levels in the YBG group were significantly reduced from baseline after 72 h (see Table 3 and all figures). Additionally, significant main effects for time (both groups changed in a similar direction and magnitude) were realized for IFNγ (*p* = 0.02), IL-6 (*p* < 0.001), IL-8 (*p* < 0.001), MCP-1 (*p* < 0.001), and MIP-1β (*p* = 0.04). As seen in Table 3, cytokine levels increased sharply in comparison to pre-exercise levels and peaked immediately post-exercise in both groups except for MIP-1β. In this respect, MIP-1β levels peaked at the two-hour time point and began returning to baseline levels by the 72-hour time point. Across the 72-hour measurement window and in those chemokines that exhibited a significant change (MIP-1β) or a tendency to change (IL-8, MCP-1, and TNF-α), YBG levels were lower when compared to PLA (See Figure 3, Figure 4, Figure 5 and Figure 6 and Table 2). To identify differences at these time points in these groups, paired samples *t*-tests using the delta scores (minus pre-exercise) revealed statistically significant reductions after 72 h in IL-8 (*p* = 0.044, 95% Confidence Interval (CI): (0.013, 0.938, *d* = −0.34), MIP-1β (*p* = 0.01, 95% CI: (0.13, 0.85), *d* = −0.57), and MCP-1 (*p* = 0.038, 95% CI: 0.087, 2.942, *d* = −0.33).

### 3.3. Complete Blood Counts

Prior to and in response to the exercise stress, complete blood counts were assessed from the collected venous blood. No significant (*p* > 0.05) group x time interaction effects were observed for any of the measured outcomes. As expected, and as a strong indication of the level of physiological stress invoked by our exercise bout, several white blood cell count variables (i.e., neutrophils, lymphocytes, monocytes, eosinophils, basophils) experienced significant (*p* < 0.001) changes across the 72-hour measurement window with no differences between the two supplement groups. All data are provided in supplementary Table S1.

### 3.4. Comprehensive Metabolic Panel

Prior to and in response to the exercise stress, comprehensive metabolic panels were completed. No significant (*p* > 0.05) group x time interaction effects were observed for any of the measured outcomes. As expected, and in a manner dependent upon the individual marker, significant within-group changes were observed in response to the exercise bout. However, all changes were within clinically published references ranges. All data are available in supplementary Table S2.

### 3.5. Muscle Damage

**Figure 7:**
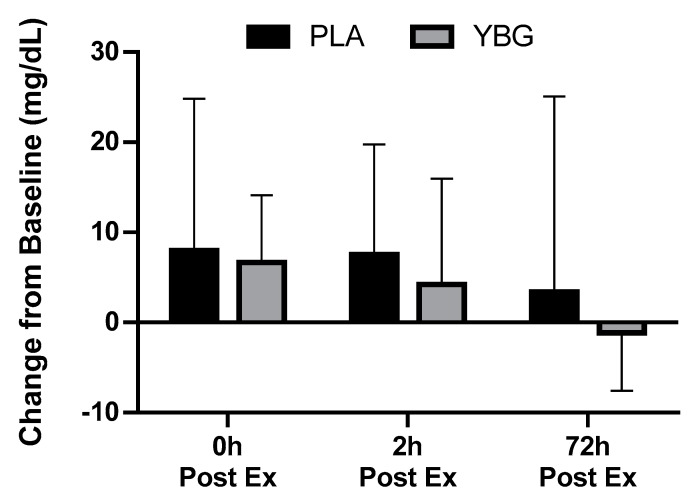
Changes in myoglobin from baseline for YBG and PLA after 13 days of supplementation and heated treadmill exercise. Data are presented as means ± SD. The 4 × 2 mixed factorial ANOVA revealed a group × time interaction effect of *p* = 0.62. Changes from baseline were calculated and compared using paired samples t-test. At the 72-hour time point, the changes observed in YBG were not significantly different (mean difference ± SD: 4.56 ± 21.91, 95% CI on difference: (−3.48, 12.59), *p* = 0.256) than the changes observed in PLA. Percent changes from baseline for PLA were +28.3%, +26.8%, and +12.1% while % changes from baseline for YBG were +24.8%, +15.7%, and –4.4% after 0, 2, and 72 h post-exercise, respectively.

Prior to and in response to the exercise stress (0, 2, and 72 h post-exercise), three assessments were completed as indicators of muscle damage: Perceived soreness, plasma creatine kinase, and plasma myoglobin. The soreness data deviated from normality and were subsequently transformed and analyzed using square root transformations and analyzed. All data provided in Table 4 are the raw data while all associated p-values and statistical determinations used the transformed data. No significant (*p* > 0.05) group x time interaction effects were observed for any of the measured outcomes: Perceived soreness (p = 0.78), creatine kinase (*p* = 0.98), and myoglobin (*p* = 0.62). Consistent significant main effects for time were observed with all variables experiencing robust increases immediately and 2 h post-exercise, which sharply decreased by 72 h. Myoglobin changes in the PLA group tended to be increased (*p* = 0.07) immediately after exercise and these elevations reached statistical significance after two hours (Figure 7). Notably, myoglobin levels in the YBG group decreased below baseline levels after 72 h whereby myoglobin levels in PLA remained non-significantly elevated from their baseline levels (Table 4).

### 3.6. Muscle Function

Prior to and in response to the exercise stress (0, 2, and 72 h post-exercise), two tests were completed to assess muscle function in response to supplementation and the exercise stress: A maximal voluntary isometric contraction tests and a 50-repetition isokinetic muscle fatigue test with multiple variables being produced from each test. No changes in isometric peak torque (G × T, *p* = 0.79) and average isometric peak torque (G × T, *p* = 0.92) were observed. In both variables, significant main effects for time were realized (*p* < 0.001), while the pattern of change was similar between groups. No significant effects were observed for changes in peak isokinetic torque (G × T, *p* = 0.35) while changes across time in both groups tended to be similar (*p* = 0.08) (Table 5) during the 50-repetition isokinetic test. Additionally, no significant effects were observed for average peak isokinetic torque (G × T, *p* = 0.94) with a significant change across time (*p* = 0.04). Follow-up pairwise comparisons revealed no orthogonal differences. No interaction effects were found for changes in fatigue index (G × T, *p* = 0.45; time effect, *p* = 0.46). Finally, the total work completed was not different between groups (G × T, *p* = 0.69), but both groups experienced similar and significant reductions immediately after the exercise bout, which returned to baseline levels by the next measurement (Table 5).

### 3.7. Profile of Mood States

Prior to and in response to the exercise stress (0, 2, and 72 h post-exercise), the profile of mood states (POMS) was completed by all participants. A significant interaction effect (G × T, *p* = 0.04) was identified for changes in the anger subscale with levels in YBG and PLA both experiencing reductions in these values throughout the post-exercise time period (Table 6). A significant interaction effect was identified (G × T, *p* = 0.04) for changes in the vigor subscale. In this respect, YBG values remained stable while PLA was significantly lower 72 h after exercise when compared to its pre-exercise value. Total mood disturbance was significantly different (G × T, *p* = 0.007). Follow-up analyses indicated that both groups experienced significant changes from baseline with YBG values experiencing sharp, statistically significant increases (*p* < 0.05) in total mood disturbance immediately post-exercise but returned to values below baseline by 72 h post-exercise. Of the remaining subscales (tension, depression, fatigue, and confusion), no statistically significant G × T interactions were observed. Examination of within-group changes for these subscales revealed statistically significant reductions in YBG for tension, fatigue, and confusion (all *p* < 0.001) while changes in the PLA group experienced a tendency to change (*p* = 0.07) for tension and confusion (Table 6).

## 4. Discussion

We sought to examine if acute supplementation with yeast beta-glucan affected various biomarkers in response to a bout of heated treadmill exercise. Primary outcomes of interest included changes in immune function markers, muscle function, and muscle damage while secondary outcomes included changes in mood state, cell counts, and clinical safety. Findings relating to the primary outcomes were mixed, with cytokine changes in the blood indicating a statistically significant (*p* = 0.044) group x time interaction for MIP-1β and statistical trends (*p* = 0.08–0.10) for TNF-α, MCP-1, and IL-8 to be differentially expressed between groups in response to the interventions. When examined at the within-group level, YBG supplementation consistently reduced the magnitude of response, and in some instances, shortened the time course increases in cytokine expression and myoglobin concentrations relative to PLA (Figure 3, Figure 4, Figure 5 and Figure 6, Table 3). Between-group changes in muscle function and all markers of muscle damage, with the exception of myoglobin, were not different across the study protocol. Additionally, no significant between-group interactions were identified for any of the white blood cell count and metabolic panel markers. Changes in mood state profiles did differ between groups. In this respect, anger, vigor, and total mood disturbance all exhibited significant group x time interaction effects and when the main effects were decomposed, both groups exhibited significant changes across time while anger values in YBG were reduced (in comparison to pre-exercise levels) 72 h after completing the exercise bout. Vigor levels remained at baseline levels (*p* = 0.65) through the entire 72-h study protocol while vigor levels in the PLA were significantly reduced (*p* = 0.003) from baseline after 72 h (group × time, *p* = 0.04, Table 6).

In recent years, published studies have highlighted the potential for beta-glucans to impact various aspects of health. For example, previous research by Nieman [18] supplemented cyclists for two weeks with beta-glucans derived from oats before completing once daily three-hour moderate-intensity rides for three consecutive days. Using this design, oat beta-glucans exhibited no impact on respiratory infections or other changes in immune function. Interestingly, beta-glucans found in mushrooms and yeast take on a more ‘branched’ arrangement, with the uniqueness of this structure being purported to impact health and immune status. As such, previous research using yeast beta-glucans (*Saccharomyces cerevisiae*) has provided initial documentation for its ability to favorably impact immunity resulting in reductions in the number, duration, and severity of common upper respiratory tract infections [20,21,22].

Other data examining the impact of yeast-derived beta-glucans have demonstrated a 37% reduction in cold and flu symptoms after a marathon [16]. In alignment with findings from the present study, Carpenter and colleagues [4] had 60 recreationally active men and women supplement with a baker’s yeast beta-glucan for 10 days prior to completing a single bout of moderate intensity cycling in a hot and humid environment. Using a lipopolysaccharide (LPS) induction cell culture model, cytokine expression and various cell counts were increased in response to exercise and beta-glucan ingestion. Key findings from our study extended these previous findings to highlight the ability of yeast-derived beta-glucans to impact muscle function, muscle damage, and mood state in response to supplementation and exercise. Additionally, previous work by Talbott et al. [23] indicated that four weeks of ingesting beta-glucans from baker’s yeast can reduce the onset of upper respiratory tract infections, while a 2017 study by McFarlin and investigators [24] concluded that baker’s yeast beta-glucan supplementation before a 90-minute bout of heated treadmill exercise improves cytokine expression and various immune cell markers and associated antigens. Previous work using the same yeast-derived beta-glucan (Yestimun®) as in the present study at a higher dose (900 mg) in non-exercising individuals significantly reduced (in comparison to placebo) the incidence of common cold symptoms in comparison to a placebo group (*p* = 0.019). Furthermore, it was shown that supplementation with 900 mg of yeast-derived beta-glucan lowers the severity of the acute phase of upper respiratory infections [20,21,22]. The current data are some of the first published using a 250-mg dose for 10 days prior to a heated exercise challenge and align with the general notion that administration of a yeast-derived beta-glucan can aid in the body’s immunological response to physical stress. A key function of yeast beta-glucans is the ability of this ingredient to mediate antimicrobial activity. This activity may stimulate macrophages resulting in a more effective response to infections, a phenomenon termed a “trained immunity” that has been previously described on the epigenetic level [15]. In this respect, (1,3)-(1,6)-beta-D-glucans were shown to activate the M2 phenotype of macrophages which produce regulatory cytokines such as IL-10, and downregulates MIP-1 β and MCP-1 release. Both factors are produced by macrophages and activate human granulocytes such as neutrophils, eosinophils, and basophils. This activation then goes on to play a primary role in acute neutrophil-mediated inflammation. These changes can also impact the production and release of other pro-inflammatory cytokines such as IL-1, IL-6, and TNF-α from fibroblasts and macrophages. Apart from MIP-1 β, MCP-1 is a chemokine that regulates the migration and infiltration of monocytes/macrophages. The intensive production of MCP-1 is involved in negative cross-talk between adipose tissue and muscle and impairs insulin signaling via ERK1/2 activation [32]. The downregulation of MCP-1β and MIP-1 β by macrophages likely indicates that the regeneration process moved to second stage of the healing, where the presence of macrophages and other immune cells is not favorable, and that the regeneration shifted into the muscle remodeling [8]. Thus, the downregulation of MCP-1β observed in YBG after heated treadmill exercise, as well as the limited release of IL-8, might be an indication of the muscle healing process associated with decreased myoglobin levels in this group 72 h after exercise stress. Overall, these outcomes are indicative of robust physiological stress secondary to the exercise and heat exposure and a potential ability of YBG to help recovery of myoglobin levels more rapidly than PLA, but more research is needed to identify the potential for this outcome. Mood state (POMS data) changes were challenging to interpret as increases in specific subscales (anger and vigor) did not align with overall mood disturbance scores (Table 6). Subsequently, more research is needed to identify the potential for YBG to impact mood state changes in response to heat and exercise challenges. A future recommendation would be to complete assessments of mood state at more frequent intervals throughout the post-exercise period than what was completed in the present study.

Key strengths for the current study point to the randomized, double-blind, placebo-controlled, cross-over study design that was employed in addition to the strict inclusion criteria. Participants in this study were required to possess an aerobic capacity level that was at the 50th percentile for their gender and age but could also not be above the 80th percentile. As a result, the participants in this study were active and consistent exercisers but were not considered to be of an elite (or low) fitness level. This criteria was set to help ensure a homogenous sample relative to fitness status, exercise background, and the extent to which each person’s immune system was adapted to handle exercise and thermal stress invoked by this project [33], as novel exercisers may have a more exacerbated immune response to a stressful bout of exercise. Limitations of our study point towards our sample size and the lack of time points between 24 and 48 h. As can be seen throughout the time course of data outlined in our results, many values were still elevated after two hours but returned to baseline within 72 h. Thus, for individuals who exercise each day, the ability of yeast beta-glucan to bolster the immune response is established from these findings, while additional research is needed to better understand the post-exercise time course. Additionally, future research should better identify the impact of yeast beta-glucans after repeated bouts of exercise stress as several previous studies, including the present study, have examined the impact of yeast-derived beta-glucans after a single bout of exercise [16,23,24]. Mechanistic considerations for yeast beta-glucan is still not fully understood. The changes observed in our blood-based markers are likely to be influenced by other tissues and cells located ubiquitously throughout systemic circulation. A deeper investigation into intramuscular changes to cytokine changes would help researchers to understand the impact of YBG at the level of the skeletal muscle. In this respect, previous research has reported differences in interferon-γ expression after stressful exercise in both trained [4] and untrained [24] populations following beta-glucan supplementation. These findings were not realized in the present study, likely due to the inability to perform a culture-based LPS induction of cytokine expression. Additionally, other changes reported by McFarlin [16] have pointed towards the ability of YBG to impact expression of various antigens on monocytes (CD8, CD86, etc.), but our work was limited in that it did not perform any flow cytometry work, an area that would have certainly strengthened our outcomes. Nevertheless, the changes observed in MIP-1β, MCP-1, IL-8, and TNF- α all point toward a favorable ability of YBG to reduce inflammatory responses observed in response to stressful exercise. 

## 5. Conclusions

A prophylactic 13-day protocol of supplementing with 250 mg of a yeast beta-glucan led to significant reductions in MIP-1β, MCP-1, and IL-8 levels and a statistical tendency for TNF-α to be lowered following a bout of heated treadmill exercise. Muscle function changes in response to the intervention were not different between groups. No between-group differences were observed in creatine kinase while after 72 h, myoglobin levels in YBG were reduced from pre-exercise levels while the PLA remained elevated from baseline at this time point. Mood state analysis (POMS) indicated a significant improvement in vigor and reduction in anger in the YBG group in conjunction with a greater reduction in total mood disturbance in comparison to PLA. Results from this preliminary investigation highlight the need for more research to build upon these findings.

## Figures and Tables

**Table 1:** Study participant characteristics.

Variable	Gender	Mean ± SD
Age (years)	Men (*n* = 16)	29.6 ± 6.7
	Women (*n* = 15)	30.1 ± 8.9
	Total (*n* = 31)	29.9 ± 7.7
Height (cm)	Men	178.1 ± 7.2
	Women	165.6 ± 4.1
	Total	172.1 ± 8.6
Body weight (kg)	Men	83.2 ± 11.2
	Women	66.7 ± 10.0
	Total	75.2 ± 13.4
DEXA Body fat (%)	Men	21.2 ± 4.4
	Women	29.6 ± 5.3
	Total	25.3 ± 6.4
DEXA Fat mass (kg)	Men	18.0 ± 5.1
	Women	20.4 ± 6.2
	Total	19.1 ± 5.7
DEXA FFM (kg)	Men	66.2 ± 8.4
	Women	47.3 ± 5.0
	Total	57.1 ± 11.8
VO_2Peak_ (mL/kg/min)	Men	49.6 ± 5.1
	Women	38.7 ± 5.8
	Total	44.3 ± 7.7

SD, standard deviation; FFM, fat-free mass.

**Table 2:** Physiological stress among individuals (*n* = 31) who completed 55 ± 9 min of treadmill exercise in a hot (37.2 ± 1.8 °C), humid (45.2 ± 8.8%) environment.

	0 min	10 min	15 min	20 min	30 min	40 min	45 min	50 min	Final*
Heart Rate (beats/min)									
PLA	93 ± 13	---	143 ±16	---	153 ± 18	---	161 ± 17	---	167 ± 15
YBG	92 ± 16	---	143 ± 15	---	154 ± 19	---	160 ± 18	---	167 ± 19
Exercise VO_2_ (mL/kg/min)									
PLA	4.7 ± 1.5	---	23.4 ± 4.5	---	24.2 ± 4.5	---	23.5 ± 6.3	---	22.3 ± 6.6
YBG	4.9 ± 1.2	---	22.9 ± 6.0	---	24.5 ± 4.8	---	24.3 ± 4.6	---	25.2 ± 6.3
Rating of Perceived Exertion (RPE)									
PLA	7 ± 1	10 ± 2	---	11 ± 2	13 ± 2	15 ± 5	---	15 ± 3	15 ± 3
YBG	6 ± 1	9 ± 2	---	11 ± 2	13 ± 2	13 ± 2	---	15 ± 3	15 ± 3
Core Temperature (°C)									
PLA	36.5 ± 3.7	36.7 ± 3.7	---	36.9 ± 3.8	37.3 ± 3.8	37.5 ± 4.1	---	37.6 ± 4.3	37.9 ± 3.9
YBG	37.2 ± 0.3	37.4 ± 0.4	---	37.7 ± 0.4	38.1 ± 0.4	38.2 ± 0.5	---	38.5 ± 0.5	38.7 ± 0.6

All data are presented as means ± SD. SD, standard deviation; RPE, rate of perceived exertion; °C, degrees Celsius. *Final time point reflects individuals exercising 60 min or until reaching a core temperature of 39.2 °C, in which test was terminated to avoid health complications associated with hyperthermia.

**Table 3:** Plasma cytokine expression.

Variable	Group	Pre-Exercise	0 h Post-Exercise	2 h Post-Exercise	72 h Post-Exercise	Within (*p*)	Time (*p*)	G × T (*p*)
Interferon-γ (pg/mL)	**PLA**	1.36 ± 1.45	1.91 ± 3.15 +40.4%	1.31 ± 1.27 −3.7%	1.46 ± 1.44 +7.4%	0.177	0.021	0.451
	**YBG**	1.60 ± 1.90	2.11 ± 2.94 +36.5%	1.68 ± 1.63 +5.0%	1.31 ± 1.35 −18.1%	0.017		
IL-6 (pg/mL)	**PLA**	0.95 ± 1.73	1.53 ± 1.97 ^a^ +61.0%	1.06 ± 1.76 ^a,b^ +11.6%	0.66 ± 0.62 ^b^ −30.5%	<0.001	<0.001	0.697
	**YBG**	0.81 ± 0.75	1.46 ± 1.07 ^a^ +80.2%	0.99 ± 0.77 ^b^ +22.2%	0.61 ± 0.51 ^b,c^ −24.7%	<0.001		
IL-8 (pg/mL)	**PLA**	1.74 ± 1.43	2.30 ± 1.95 +32.2%	2.06 ± 1.75 +18.4%	1.84 ± 1.15 +5.8%	0.003	<0.001	0.079
	**YBG**	2.06 ± 1.66	2.37 ± 1.83 +15.0%	2.00 ± 1.36 −2.6%	1.68 ± 1.28 −18.4%	0.001		
MCP-1 (pg/mL)	**PLA**	8.52 ± 5.15	12.18 ± 7.60 +43.0%	9.43 ± 5.56 +10.7%	9.41 ± 5.42 +10.5%	<0.001	<0.001	0.095
	**YBG**	9.17 ± 4.92	12.22 ± 7.80 +33.0%	9.75 ± 5.49 +6.3%	8.55 ± 4.81 −6.8%	0.001		
MIP-1β (pg/mL)	**PLA**	1.68 ± 1.24	1.92 ± 1.60 +14.1%	2.11 ± 1.56 +25.7%	1.98 ± 1.37 +17.6%	0.043	0.039	0.044
	**YBG**	2.13 ± 1.59	2.21 ± 2.03 +3.7%	2.41 ± 1.94 +12.9%	1.94 ± 1.68 −9.1%	0.039		
TNF-α (pg/mL)	**PLA**	5.94 ± 4.31	6.38 ± 5.23 +7.4%	5.93 ± 3.87 −0.2%	6.35 ± 4.00 +6.9%	0.245	0.168	0.085
	**YBG**	6.56 ± 4.61	7.00 ± 5.43 +6.8%	6.16 ± 4.12 −6.1%	6.13 ± 4.25 −6.5%	0.043		

PLA, placebo (maltodextrin); YBG, yeast beta-glucan; pg, picograms; mL, milliliters; G × T = group × time interaction; all data presented as mean ± SD; **^a^** d a significant difference (*p* < 0.05) from pre-exercise; **^b^** denotes a significant difference (*p* < 0.05) from 0 h post-exercise; **^c^** denotes a significant difference (*p* < 0.05) from 2 h post-exercise.

**Table 4:** Indirect markers of muscle damage.

Variable	Group	Pre-Exercise	0 h Post-Exercise	2 h Post-Exercise	72 h Post-Exercise	Within (*p*)	Time (*p*)	G × T (*p*)
Perceived Soreness (mm)	**PLA**	12.9 ± 11.9	20.4 ± 17.8 ^a,b^	18.9 ± 16.2 ^a,b^	8.8 ± 6.5	<0.001	<0.001	0.78
	**YBG**	14.4 ± 16.0	19.7 ± 20.9 ^b^	19.3 ± 18.5 ^b^	9.8 ± 7.1	0.004		
Creatine Kinase (U/L)	**PLA**	108.3 ± 55.4	125.3 ± 64.5 ^a^	125.9 ± 64.5 ^a^	87.9 ± 44.6 ^b,c^	<0.001	<0.001	0.72
	**YBG**	106.8 ± 64.3	123.1 ± 74.9 ^a^	125.8 ± 69.8 ^a^	87.2 ± 42.4 ^b,c^	<0.001		
Myoglobin (mg/dL)	**PLA**	28.1 ± 32.4	36.1 ± 34.9	35.7 ± 37.1 ^a^	31.5 ± 39.4	0.07	<0.001	0.62
	**YBG**	26.8 ± 30.7	33.4 ± 34.6 ^a^	31.0 ± 24.1	25.6 ± 26.4 ^b,c^	0.001		

PLA, placebo (maltodextrin); YBG, yeast beta-glucan; all data presented as mean ± SD; U, units; L, liters; mg, milligrams; dL, deciliters; ^a^ denotes a significant difference (*p* < 0.05) from pre-exercise; ^b^ denotes a significant difference (*p* < 0.05) from 0 h post-exercise; ^c^ denotes a significant difference (*p* < 0.05) from 2 h post-exercise. ANOVA models for perceived soreness were completed using square root transformation while natural logarithmic transformations were used for creatine kinase data due to violations of normality assumptions. Raw data are entered in above table.

**Table 5:** Muscle function.

Variable	Group	Pre-Exercise	0 h Post-Exercise	2 h Post-Exercise	72 h Post-Exercise	Within (*p*)	Time (*p*)	G × T (*p*)
Isometric Peak Torque (N·m)	**PLA**	171.5 ± 48.9	158.1 ± 47.7 ^a^	159.0 ± 52.0 ^a^	167.9 ± 50.1	<0.001	<0.001	0.79
	**YBG**	165.9 ± 56.8	151.2 ± 46.0	152.8 ± 46.3 ^b^	165.0 ± 49.5 ^b, c^	0.003		
Isometric Average Peak Torque (N·m)	**PLA**	164.7 ± 47.8	148.1 ± 43.6 ^a^	150.8 ± 48.8 ^a^	160.1 ± 49.5 ^b, c^	<0.001	<0.001	0.92
	**YBG**	160.6 ± 46.6	142.9 ± 45.6 ^a^	146.7 ± 45.6 ^a^	157.1 ± 47.7 ^b, c^	<0.001		
Isokinetic Peak Torque (N·m)	**PLA**	136.9 ± 44.6	126.2 ± 40.7 ^a^	126.7 ± 43.0 ^a^	129.1 ± 44.5 ^a^	<0.001	0.08	0.35
	**YBG**	132.5 ± 48.3	128.7 ± 44.8	125.5 ± 44.3 ^a^	123.6 ± 50.5	0.19		
Isokinetic Average Peak Torque (N·m)	**PLA**	87.4 ± 29.6	84.5 ± 29.9	86.3 ± 31.9	86.5 ± 30.9	0.07	0.04	0.94
	**YBG**	86.5 ± 31.1	84.5 ± 31.5	85.3 ± 32.1	85.9 ± 31.5	0.20		
Fatigue Index (%)	**PLA**	48.9 ± 10.9	45.7 ± 11.2	45.5 ± 13.1	46.9 ± 15.0	0.69	0.46	0.45
	**YBG**	48.5 ± 12.7	46.5 ± 14.2	44.2 ± 14.5	47.2 ± 11.5	0.07		
Total Work Completed (J)	**PLA**	5176 ± 1371	4749 ± 1363 ^a^	5009 ± 1491	5172 ± 1689 ^b^	0.05	0.001	0.69
	**YBG**	5257 ± 1665	4912 ± 1496 ^a^	5039 ± 1566	5176 ± 1607 ^b^	<0.001		

PLA, placebo (maltodextrin); YBG = yeast beta-glucan; G × T = group x time interaction; all data presented as mean ± SD; N·m, Newtons/meters; %, percent (%) change of work from final third of test bout compared to work completed during the first third of the test bout; J, Joules; ^a^ denotes a significant difference (*p* < 0.05) from pre-exercise; ^b^ denotes a significant difference (*p* < 0.05) from 0 h post-exercise; ^c^ denotes a significant difference (*p* < 0.05) from 2 h post-exercise.

**Table 6:** Profile of mood states (POMS).

Variable	Group	Pre-Exercise	0 h Post-Exercise	2 h Post-Exercise	72 h Post-Exercise	Within Group	Time (*p*)	G × T (*p*)
Tension	**PLA**	14.2 ± 4.2	13.8 ± 3.1	12.9 ± 3.3 ^b^	13.3 ± 4.0	0.07	<0.001	0.24
	**YBG**	15.4 ± 4.9	15.4 ± 5.1	14.2 ± 4.8 ^a,b^	13.6 ± 4.4 ^a,b^	<0.001		
Depression	**PLA**	18.4 ± 7.0	17.9 ± 6.3	17.3 ± 5.4 ^a,b^	17.7 ± 5.8	0.009	0.01	0.17
	**YBG**	18.4 ± 5.1	18.5 ± 5.6	18.1 ± 5.7b	17.6 ± 4.6	0.15		
Anger	**PLA**	14.8 ± 3.6	13.6 ± 2.6 ^a^	13.7 ± 3.1	13.5 ± 3.0 ^a^	0.001	<0.001	0.04
	**YBG**	16.0 ± 4.2	15.5 ± 4.4	14.4 ± 3.8 ^a^	14.2 ± 3.1 ^a^	<0.001		
Vigor	**PLA**	25.6 ± 7.5	24.8 ± 7.0	24.8 ± 7.1	23.1 ± 7.9 ^a^	0.003	0.09	0.04
	**YBG**	24.7 ± 5.8	23.9 ± 6.6	24.5 ± 6.8	24.6 ± 6.1	0.65		
Fatigue	**PLA**	12.4 ± 5.3	14.4 ± 5.9	12.7 ± 4.7	12.1 ± 5.0 ^b^	0.01	0.001	0.12
	**YBG**	13.1 ± 5.1	15.3 ± 5.1	13.1 ± 4.0 ^b^	11.2 ± 3.4 ^b^	<0.001		
Confusion	**PLA**	11.4 ± 3.4	10.7 ± 3.2	10.5 ± 2.7 ^a^	10.5 ± 2.8	0.07	0.004	0.30
	**YBG**	11.4 ± 2.8	11.4 ± 3.1	10.6 ± 2.6	10.4 ± 2.7 ^a,b^	0.001		
Total Mood Disturbance	**PLA**	45.6 ± 23.3	45.6 ± 20.1	42.2 ± 19.2	44.0 ± 20.9 ^a,b^	0.001	<0.001	0.007
	**YBG**	49.5 ± 21.5	52.2 ± 21.5	45.9 ± 21.3 ^b^	42.4 ± 18.6	0.015		

PLA, placebo (maltodextrin); YBG = yeast beta-glucan; G × T = group × time interaction; all data presented as mean ± SD; ^a^ denotes a significant difference (*p* < 0.05) from pre-exercise; ^b^ denotes a significant difference (*p* < 0.05) from 0 h post-exercise; ^c^ denotes a significant difference (*p* < 0.05) from 2 h post-exercise.

## Supplementary Materials

The following are available online at https://www.mdpi.com/2072-6643/12/4/1144/s1, Table S1, Complete blood counts with platelet differentials; Table S2: Comprehensive metabolic panel.
