# Supplementary material for: Yeast Beta-Glucan Supplementation Downregulates Markers of Systemic Inflammation after Heated Treadmill Exercise
Source: Nutrients. 2020 Apr 19;12(4):1144. doi: 10.3390/nu12041144 (PMC7230631; doi:10.3390/nu12041144)
Supplement: Supplementary file 1 [file nutrients-12-01144-s001.pdf]

Table S1: Complete Blood Counts with Platelet Differentials.

Variable	Group	Pre-Exercise	0 h Post-Exercise	2 h Post-Exercise	72 h Post-Exercise	Within (p)	Time (p)	G x T (p)
WBC Count (Thousand/μL)	**PLA**	6.03 ± 1.86	7.04 ± 2.25	7.68 ± 2.06	5.72 ± 1.33	< 0.001	< 0.001	0.69
	**YBG**	5.73 ± 1.32	6.96 ± 1.81	7.65 ± 2.03	5.75 ± 1.52	< 0.001		
RBC Count (Million/μL)	**PLA**	4.74 ± 0.42	4.81 ± 0.46	4.75 ± 0.38	4.74 ± 0.42	0.264	0.15	0.62
	**YBG**	4.66 ± 0.66	4.81 ± 0.49	4.77 ± 0.47	4.69 ± 0.37	0.248		
Hemoglobin (g/dL)	**PLA**	14.6 ± 1.2	14.8 ± 1.4	14.6 ± 1.1	14.4 ± 1.2	0.068	0.005	0.65
	**YBG**	14.7 ± 1.2	14.8 ± 1.4	14.7 ± 1.3	14.4 ± 1.1	0.041		
Hematocrit (%)	**PLA**	43.2 ± 3.2	43.5 ± 3.7	43.1 ± 2.9	43.0 ± 3.1	0.683	0.34	0.83
	**YBG**	43.5 ± 3.2	43.5 ± 3.6	43.1 ± 3.7	43.0 ± 2.9	0.384		
MCV (fL)	**PLA**	91.2 ± 3.0	90.5 ± 3.0 [a]	90.8 ± 2.9	91.2 ± 3.2	0.039	0.001	0.28
	**YBG**	91.5 ± 3.3	90.7 ± 3.1 [a,b]	90.6 ± 3.1 [a]	91.7 ± 3.1	0.003*		
MCH (pg)	**PLA**	30.8 ± 1.3	30.8 ± 1.2	30.7 ± 1.2	30.6 ± 1.3	0.138	0.10	0.81
	**YBG**	30.9 ± 1.3	30.9 ± 1.3	30.9 ± 1.2	30.8 ± 1.1	0.578		
MCHC (g/dL)	**PLA**	33.8 ± 0.8	34.1 ± 0.7	33.8 ± 0.8	33.5 ± 1.0	0.027	0.001	0.36
	**YBG**	33.8 ± 1.0	34.1 ± 0.9	34.1 ± 0.8	33.6 ± 1.0	0.011		
RDW (%)	**PLA**	12.3 ± 0.5	12.4 ± 0.5	12.3 ± 0.5	12.4 ± 0.5	0.181	0.04	0.72
	**YBG**	12.3 ± 0.5	12.3 ± 0.4	12.2 ± 0.4	12.3 ± 0.5	0.122		
Platelet Count (Thousand/μL)	**PLA**	242.1 ± 62.2	288.4 ± 74.4	239.3 ± 60.2	234.9 ± 62.2	< 0.001	< 0.001	0.50
	**YBG**	241.1 ± 54.1	286.7 ± 63.8	246.3 ± 53.3	238.5 ± 55.2	< 0.001		

Neutrophils (cells/μL)	**PLA**	3094 ± 1350	4190 ± 1904	5272 ± 1852	2907 ± 847	< 0.001	< 0.001	0.83
	**YBG**	2890 ± 795	4206 ± 1412	5244 ± 1861	2945 ± 918	< 0.001		
Lymphocytes (cells/μL)	**PLA**	2139 ± 818	2138 ± 752	1699 ± 444 [a,b]	2189 ± 738	< 0.001	< 0.001	0.57
	**YBG**	2110 ± 712	2024 ± 554	1721 ± 451 [a,b]	2198 ± 738	< 0.001		
Monocytes (cells/μL)	**PLA**	491.0 ± 203.6	542.1 ± 243.9 [a]	547.6 ± 244.3	484.2 ± 115.8	0.048*	< 0.001	0.64
	**YBG**	454.1 ± 106.2	500.8 ± 115.4	533.4 ± 148.9 [a,b]	456.3 ± 109.3	0.002*		
Eosinophils (cells/μL)	**PLA**	194.6 ± 141.1	163.7 ± 134.8 [a,b]	113.3 ± 91.1 [a,b]	211.9 ± 143.6	< 0.001	< 0.001	0.65
	**YBG**	213.7 ± 154.4	174.5 ± 138.6 [a]	128.0 ± 102.4 [a,b]	208.9 ± 137.0	< 0.001		
Basophils (cells/μL)	**PLA**	46.9 ± 24.2	56.3 ± 29.5 [a]	43.4 ± 22.6	48.8 ± 22.9	< 0.001	< 0.001	0.99
	**YBG**	47.4 ± 23.8	57.6 ± 28.6 [a]	46.5 ± 25.9	50.6 ± 26.4	0.005		
Neutrophils (%)	**PLA**	51.3 ± 10.1	57.5 ± 10.0	67.2 ± 7.9	50.4 ± 8.7	< 0.001	< 0.001	0.61
	**YBG**	50.2 ± 7.5	58.7 ± 8.6	66.8 ± 7.8	50.1 ± 7.7	< 0.001		
Lymphocytes (%)	**PLA**	36.4 ± 10.1	31.7 ± 9.8 [a,b]	23.5 ± 7.1 [a,b]	37.1 ± 8.5	< 0.001	< 0.001	0.56
	**YBG**	36.9 ± 7.8	29.7 ± 6.9 [a,b]	23.7 ± 6.8 [a,b]	37.5 ± 7.9	< 0.001		
Monocytes (%)	**PLA**	8.09 ± 2.15	7.66 ± 1.91	7.10 ± 1.85	8.50 ± 1.97	< 0.001	< 0.001	0.22
	**YBG**	8.21 ± 2.28	7.45 ± 1.88	7.12 ± 1.71	7.95 ± 2.06	0.001		
Eosinophils (%)	**PLA**	3.19 ± 2.15	2.32 ± 1.75 [a,b]	1.63 ± 1.35 [a,b]	3.52 ± 2.07	< 0.001	< 0.001	0.41
	**YBG**	3.57 ± 2.13	2.47 ± 1.68 [a,b]	1.70 ± 1.22 [a,b]	3.52 ± 2.15	< 0.001		
Basophils (%)	**PLA**	0.77 ± 0.32	0.79 ± 0.32	0.57 ± 0.27	0.84 ± 0.35	< 0.001	< 0.001	0.74
	**YBG**	0.84 ± 0.36	0.83 ± 0.39	0.63 ± 0.32	0.85 ± 0.36	< 0.001		

PLA = Placebo (Maltodextrin); YBG = Yeast Beta-Glucan; μL = Microliters; g = grams; dL = deciliter; pg = picograms; WBC = white blood cell; RBC = red blood cell; MCV = Mean corpuscle volume; MCH = Mean corpuscle hemoglobin; MCHC = Mean corpuscle hemoglobin content; RDW = Red cell dimension width; Abs = Absolute. G x T =

group x time interaction. All data presented as Mean ± SD. [a] Denotes a significant difference ($p < 0.05$) from Pre-Exercise; [b] Denotes a significant difference ($p < 0.05$) from 0 h Post-Exercise; [c] Denotes a significant difference ($p < 0.05$) from 2 h Post-Exercise. .

Table S2. Comprehensive Metabolic Panel.

Variable	Group	Pre-Exercise	0 h Post-Exercise	2 h Post-Exercise	72 h Post-Exercise	Within (*p*)	Time (*p*)	G x T (*p*)
Glucose (mg/dL)	**PLA**	91.2 ± 10.6	102.7 ± 15.5	89.7 ± 6.4	91.4 ± 7.9	< 0.001	< 0.001	0.845
	**YBG**	92.9 ± 9.2	103.3 ± 15.8	90.0 ± 7.0	93.4 ± 10.4	< 0.001		
BUN (mg/dL)	**PLA**	15.3 ± 4.3	15.2 ± 4.1	14.3 ± 3.8	16.0 ± 4.3	0.006	< 0.001	0.369
	**YBG**	15.3 ± 4.1	15.3 ± 3.9	14.4 ± 3.6	16.9 ± 4.4	0.005		
Creatinine (mg/dL)	**PLA**	0.88 ± 0.14	1.00 ± 0.2	0.91 ± 0.17	0.90 ± 0.14	< 0.001	< 0.001	0.983
	**YBG**	0.88 ± 0.14	0.99 ± 0.18	0.92 ± 0.15	0.89 ± 0.15	< 0.001		
eGFR (mL/min/1.73m[2])	**PLA**	104.1 ± 13.5	91.0 ± 13.8	100.4 ± 14.1	101.7 ± 13.8	< 0.001	< 0.001	0.519
	**YBG**	102.5 ± 11.7	89.5 ± 11.5	99.7 ± 10.7	102.6 ± 13.9	< 0.001		
BUN/Creatinine Ratio	**PLA**	17.4 ± 4.4	15.5 ± 4.3 [a,b]	15.8 ± 3.9 [a,b]	17.9 ± 4.2	< 0.001	< 0.001	0.370
	**YBG**	17.4 ± 3.7	15.6 ± 3.8 [a,b]	15.8 ± 3.3 [a,b]	19.1 ± 4.7	< 0.001		
Sodium (mmol/L)	**PLA**	138.3 ± 1.7	137.0 ± 1.8	135.8 ± 2.0	139.9 ± 1.7	< 0.001	< 0.001	0.384
	**YBG**	137.9 ± 2.3	137.0 ± 2.5	135.9 ± 2.1	139.3 ± 1.7	< 0.001		
Potassium (mmol/L)	**PLA**	4.41 ± 0.35	4.34 ± 0.20	4.49 ± 0.32	4.48 ± 0.37	0.114	0.003	0.821
	**YBG**	4.45 ± 0.33	4.35 ± 0.24	4.57 ± 0.37	4.51 ± 0.34	0.010		
Chloride (mmol/L)	**PLA**	102.9 ± 2.1	101.7 ± 2.0	100.0 ± 2.6	104.3 ± 2.2	< 0.001	< 0.001	0.683
	**YBG**	102.8 ± 2.5	101.6 ± 1.9	99.8 ± 2.6	103.7 ± 2.3	< 0.001		

Carbon Dioxide (mmol/L)	**PLA**	26.3 ± 2.5	23.3 ± 2.3	25.7 ± 2.1	26.8 ± 2.3	< 0.001	< 0.001	0.39
	**YBG**	26.2 ± 2.3	22.9 ± 2.5	25.9 ± 2.3	27.3 ± 2.7	< 0.001		
Calcium (mg/dL)	**PLA**	9.41 ± 0.47	9.73 ± 0.49	9.73 ± 0.51	9.42 ± 0.43	< 0.001	< 0.001	0.51
	**YBG**	9.36 ± 0.39	9.73 ± 0.45	9.76 ± 0.48	9.46 ± 0.37	< 0.001		
Total Protein (g/dL)	**PLA**	6.77 ± 0.59	7.09 ± 0.58	7.05 ± 0.62	6.65 ± 0.58	< 0.001	< 0.001	0.31
	**YBG**	6.72 ± 0.52	7.17 ± 0.60	7.03 ± 0.62	6.71 ± 0.51	< 0.001		
Albumin (g/dL)	**PLA**	4.37 ± 0.32	4.64 ± 0.39	4.61 ± 0.40	4.34 ± 0.39	< 0.001	< 0.001	0.16
	**YBG**	4.31 ± 0.32	4.63 ± 0.41	4.60 ± 0.39	4.39 ± 0.33	< 0.001		
Globulin (g/dL)	**PLA**	2.40 ± 0.40	2.45 ± 0.35	2.45 ± 0.40	2.31 ± 0.34	0.001	< 0.001	0.44
	**YBG**	2.42 ± 0.34	2.53 ± 0.38	2.44 ± 0.38	2.32 ± 0.32	< 0.001		
Alb/Glob Ratio	**PLA**	1.87 ± 0.30	1.93 ± 0.30	1.93 ± 0.33	1.93 ± 0.39	0.373	0.03	0.58
	**YBG**	1.81 ± 0.27	1.87 ± 0.30	1.92 ± 0.31	1.93 ± 0.28	0.011		
Total Bilirubin (mg/dL)	**PLA**	0.60 ± 0.34	0.75 ± 0.43 [a,b]	0.88 ± 0.53 [a,b]	0.58 ± 0.35	< 0.001†	< 0.001	0.24
	**YBG**	0.62 ± 0.31	0.78 ± 0.38 [a,b]	0.92 ± 0.47 [a,b]	0.55 ± 0.32	< 0.001†		
Alkaline Phosphatase (U/L)	**PLA**	55.9 ± 19.9	57.8 ± 19.8	55.1 ± 17.9	55.8 ± 18.7	0.129	0.02	0.32
	**YBG**	52.4 ± 13.6	55.3 ± 14.5	54.8 ± 15.3	53.4 ± 17.5	0.071		
AST (U/L)	**PLA**	19.6 ± 5.7	21.9 ± 6.0 [a,b]	21.6 ± 5.9 [a,b]	18.1 ± 4.6	< 0.001†	< 0.001	0.85
	**YBG**	19.7 ± 6.0	22.1 ± 6.4 [a,b]	21.9 ± 6.0 [a,b]	18.4 ± 5.4	< 0.001†		
ALT (U/L)	**PLA**	15.2 ± 7.7	16.2 ± 8.3	16.3 ± 8.3	14.9 ± 6.9	0.068†	< 0.001	0.35
	**YBG**	15.6 ± 6.6	17.2 ± 7.3 [a,b]	17.4 ± 6.7	15.3 ± 7.0	0.015†		

PLA = Placebo (Maltodextrin); YBG = Yeast Beta-Glucan; eGFT = Estimated glomerular filtration rate; BUN = Blood urea nitrogen; Alb = albumin; Glob = Globulin; AST = aspartate aminotransferase; ALT = alanine aminotransferase; mg = milligrams; dL = deciliter; mL = milliliters; min = minutes; m^2 = meters squared; mmol = millimoles; L = liters; g = grams; U = units. G x T = group x time interaction. All data presented as Mean ± SD. [a] Denotes a significant difference ($p < 0.05$) from Pre-Exercise; [b] Denotes a significant difference ($p < 0.05$) from 0 h Post-Exercise; [c] Denotes a significant difference ($p < 0.05$) from 2 h Post-Exercise.
